# Geographic variation and determinants of help seeking behaviour among married women subjected to intimate partner violence: evidence from national population survey

**DOI:** 10.1186/s12939-020-01355-5

**Published:** 2021-01-06

**Authors:** Muluken Dessalegn Muluneh, Yeshemebet Worku Alemu, Maereg Wagnew Meazaw

**Affiliations:** 1 Monitoring Evaluation and Research Department, Amref Health Africa in Ethiopia, Addis Ababa, Ethiopia; 2 School of Nursing and Midwifery, Western Sydney University, Sydney, Australia; 3 Faculty of Public Health, GAMBY College of Medical Science, Addis Ababa, Ethiopia; 4 School of Medicine and Public Health, Faculty of Health and Medicine, University of Newcastle, Newcastle, Australia

**Keywords:** Intimate partner violence, Married women, Geographic, Hierarchical logistic, DHS, Ethiopia

## Abstract

**Background:**

Help seeking behaviour amongst married women who experienced Intimate Partner Violence (IPV) has received limited attention in Africa. This study examines the geographic variation and investigates determinants of help seeking behaviour amongst married women in Ethiopia.

**Methods:**

This study analysed data from the 2016 Ethiopian Demographic and Health Survey (EDHS). Data was extracted for married women age 15–49 years old who experienced IPV. Factors associated with help seeking behaviour were identified using multiple logistic regression adjusted for clustering and weighing. The weighted proportion of factors associated with help seeking behaviour was exported to ArcGIS to conduct autocorrelation analysis.

**Results:**

The prevalence of help seeking behaviour among married women who experienced IPV was 19.8% (95% CI: 15.9–24.3%). Only 9.2% of them sought help from a formal source (such as police, lawyer or doctor). Multiple logistic regression analyses showed physical violence (Adjusted odds ratio (AOR)=2.76), educational attainment (AOR=2.1), a partner’s alcohol consumption (AOR=1.9), partner’s controlling behaviour (AOR= 2.4), partner’s employment status, (AOR**=** 1.9) and wealth index (AOR=2.8) were significantly associated factors with help seeking behaviour among married women who experienced IPV in Ethiopia (*P*< 0.05). Women in Benishangul-Gumuz, Gambella, Harari, Western and Eastern Amhara, and Afar had the lowest odds of help seeking behaviour (*P*< 0.001) after experiencing IPV.

**Conclusion:**

The findings of this study suggest that poor help seeking behaviour for married women experiencing IPV is a significant public health problem in Ethiopia. Multiple interrelated factors were associated with poor help seeking behaviour. These factors include women’s level of educational attainment, women experiencing physical violence, partners exhibiting controlling behaviour, partner’s alcohol consumption, the employment status of the partner, and wealth status of the household were important predictors of help seeking behaviour. Policies and interventions need to be tailored to address these factors to improve women’s health outcomes and to prevent IPV.

## Introduction

The World Health Organization (WHO) defines intimate partner violence (IPV), as “any behaviour within an intimate relationship that causes physical, psychological or sexual harm to those in the relationship that includes acts of physical aggression, psychological abuse, sexual coercion, and controlling behaviours” [1]. Globally, 30% of women experience gender based violence (GBV) [2]. In Sub-Saharan Africa, the prevalence of GBV exceeds the global rate at 44% prevalence [3]. IPV is the most common form of GBV, accounting for 75 to 85% of violence against women globally [4, 5]. IPV has been declared a universal problem across all contexts and countries, but distribution of occurrence widely varies [5–7]. A recent nationwide survey in Ethiopia revealed the lifetime prevalence of any form of IPV (physical, emotional or sexual) was 34% [8]. More broadly, 57% of married women reported experiencing at least one form of controlling behaviour from their partner [8].

In addition to the high prevalence of IPV globally, the prevalence of help seeking behaviour reported by women who have experienced IPV is low [9, 10]. The WHO’s multi-country study of IPV reported that 55–95% of women who had experienced physical or sexual IPV have never sought help from a formal institution [5, 11, 12]. The literature suggests women who have experienced IPV rarely report their experiences to formal sources and many do not seek any form of support [10]. The prevalence of help-seeking and disclosure behaviour, however, varies widely between countries [5, 11, 13, 14].

Studies show a clear pattern in which gender equity in a society influences the help seeking behaviour of women. Women in low-income countries with large gender inequality and rigid gender roles seek less help than women in countries with higher levels of gender equality, where gender roles are fluid [15, 16]. In Ethiopia and other SSA countries, rigid gender roles are widespread. Patriarchal values that emphasize male authority in several aspects of everyday life are deep-rooted. This patriarchal culture enforces male dominance and enables controlling and violent behavior [9, 16–18].

Although Ethiopia has set various legal declarations and policies that support gender equality, rigid gender roles and inequity remains rampant. These governmental declarations include the 1994 Constitution that promotes equality of men and women [19]; the 2000 Revised Family Law on marriage and resource sharing [20], and the 2004 Penal Code of Ethiopia to combat violence against women [21]. In addition, Ethiopia has signed international and continental agreements and criminal codes that address acts of violence against women. In spite the legal measures and policy enactment, IPV is still widespread and help seeking behaviour by women in Ethiopia remains poor. A large majority of existing programs and policies target survivors who have already accessed services. There is limited research focused on the help seeking behaviour of IPV survivors in developing countries, including Ethiopia. To better understand the hidden nature of violence and to better reach women experiencing IPV, it is critical the rate and patterns of reporting, and the determinants of help seeking behaviour are explored. Therefore, this study aims to conduct geospatial analysis and to investigate associated factors of help seeking behaviour among married women of reproductive age between 15 to 49 years old in Ethiopia.

## Methods

### Study setting, design and data source

Ethiopia is the second most populous country in Africa. Located in Eastern SSA [22]. Ethiopia has one of the lowest gender equality performance indicators in SSA countries [22]. Women and girls in Ethiopia are strongly disadvantaged in the areas of literacy, health, livelihood, and basic human rights [22]. They have the lowest socioeconomic status among the population and lack adequate social support networks [23–26].

This study uses data from the 2016 Ethiopian Demographic and Health Survey (EDHS). The 2016 EDHS survey is the fourth population cross sectional survey to be conducted by the Ethiopian Central Statistical Agency (CSA). The survey was conducted in nine administrative regional states, Tigray, Afar, Amhara, Oromia, Somali, Benishangul-Gumuz, Southern Nation and Nationalities, Harari and Gambella, and two administrative cities (Addis Ababa and Dire Dawa). During the survey collection period, all individuals who spent the night in that particular area were interviewed (defacto data collection method) during door to door survey [23]. The 2016 EDHS was the first ever to include a module of questions related to domestic violence. According to the ethical guidelines of the WHO, single women were randomly selected from each household to complete the survey [27].

### Data extraction and instrument

The EDHS employed standard questionnaires developed by the MEASURE DHS program. The survey datasets were downloaded from the MEASURE DHS website [28] in STATA format with permission. Since the domestic violence data was not collected in the EDHS 2000, EDHS 2005 and EDHS 2011, only 2016 EDHS data was considered for this study. After reviewing the detailed datasets and coding, further data recoding took place. All potential factors including individual, relationship, community, and social determinants were extracted from the datasets.

### Sample size and sampling procedure

Sampling for this study was based on standard EDHS sampling techniques. The 2016 EDHS used 84,915 enumeration areas; each enumeration area has an average of 181 households, from nine regions and two city administrations. Then, two stage stratified cluster sampling was used. First, 645 enumeration areas were selected from urban (202 enumeration areas) and rural (443 enumeration areas) areas based on proportional size allocation. In the second stage, on average, 28 households were selected per enumeration area using systematic random sampling. For the violence against women module, only one woman per household was interviewed. Overall, 5860 women were interviewed about their experiences of GBV with a response rate of 97% [23]. A total of 4720 married women responded to the spousal violence questionnaire. Only married women who had an experience of violence (*n*=1423) were included in this study to assess their help seeking behaviour.

### Variables

This study assesses help seeking behaviour (Yes/No) as the dependent variable. This help seeking behaviour was examined against four levels of independent variables including individual variables (women characteristics), partner/ family characteristics, community level, and societal level variables. A literature review conducted on associated factors in SSA and the EDHS datasets were used to identify and select the variable categories presented in Table 1.

**Table 1:** Variables for identifying determinates of help seeking among married women

Variable label	Variable name in the dataset
Dependent variable: Help seeking	It takes a binary form (1 = Yes help seeking) or failure (0 =No help seeking)
1.Individual / Women characteristics	
Maternal age	Mother’s age (1 = 15–24; 2 = 25–34; 3 = 35–49)
Maternal religion	Maternal religion (1 = Islam; 2 = Orthodox; 3 = catholic or others including traditional religion)
Maternal working status	Maternal working status (1 = working; 2 = not working)
Highest educational qualification	Mother education (1=Secondary and above; 2= Primary; 3= No education)
Women’s age at first marriage	Women’s age at first marriage (1=< 18; 2=18–24; 3=25–34; 4=35–49 years old)
Women drank alcohol	Women drinks alcohol (0 = No, 1 =Yes)
Access to communication media	Access to at least one of communication (1 =Yes; 2 =No)
Currently marital status	Currently marital status (1 = Currently married, 2 =Formerly married)
Physical violence experience	Physical violence experience (0 =No, 1 =Yes)
Sexual violence experience	Sexual violence experience (0 =No, 1 =Yes)
Emotional violence experience	emotional violence experience (0 =No, 1 =Yes)
2. Partner/Family characteristics	
Age of husband	Husband/ Partner age (1 =15–24; 2= 25–35; 3 =36–50; 4 => 50)
Duration of Marriage with current partner	Duration of Marriage (1 =0–4 Yrs; 2=5–9 Yrs; 3= 10 Yrs and Above)
Total number of children	Number of children (1 =0–2; 2 =3–5; 3 = > 5)
Current husband working status	Current husband working status(1=Working; 2 =not working)
Husband education qualification	Husband education (1=Secondary and above; 2= Primary; 3= No education)
Controlling behaviour reasoned out	Controlling behaviour mentioned (0 = No; 1=Yes)
History of family beating	History of family beating (0 = No; 1=Yes)
Partner drinks alcohol	Partner drinks alcohol (0 = No; 1=Yes)
3. Community characteristics	
Place of residence	Place of residence (1= Urban; 2 rural)
Geographical admin of the country	Region (1=Tigray; 2=Afar; 3= Amhara; 4= Oromia; 5=Somali; 6= Benishangul-Gumuz; 7= SNNP; 8=Gambella; 9=Harari; 10= Addis Ababa; 11= Dire Dewa)
4. Societal characteristics	
Wealth index	Wealth (1 = Rich; 2 = Middle; 3= poor)
Women decision making index	Decision power(1=Full power; 2=Considerable power; 3=No Power for decision making)
Attitude to wife beating	Justified for any reason (0 = No; 1=Yes)

### Analysis plan

Both descriptive and multivariable logistic regression analysis was conducted. As recommended by Hosmer and Lemeshow, statistically significant variables at *p*-value < 0.25 during bivariate logistic regression analysis were used for the multivariable logistic regression analysis [29]. A staged multivariable logistic regression adjusted for clustering and sampling weights was conducted. The multivariate analysis was conducted to identify the independent effect of the various independent variables on help seeking behaviour after controlling for other factors that include individual, partner, community, and societal factors (Table 2).

**Table 2:** Potential covariates used for hierarchical logistic regression model of help seeking behaviour

Model 1	Model 2	Model 3	Model 4
Women’s education, marital status, women drank alcohol, women’s access to communication media, physical violence experience emotional violence	Model 1 variables, age of partner, number of children, partner’s employment status, Partner’s education, Partner exerted controlling behaviour, history of woman’s father being physically violent towards her mother and drank alcohol	Model 2 variables, place of residence, region	Model 3 variables, wealth index, Women decision making index, attitude to wife beating

The odds ratios (ORs) and 95% confidence intervals (CIs) were calculated to assess the adjusted risk factors that affect the study’s outcome. Those with *p* < 0.05 were retained in the final model.

### Spatial analysis

Help seeking behaviour data was exported to ArcGIS 10.3.4 to visualize estimations. Geospatial analysis was conducted to determine whether the help seeking behaviour was significantly clustered or randomly clustered in particular locations of Ethiopia. Spatial autocorrelation in the prevalence of help seeking behaviour was measured using the Global Moran’s I statistic [30]. The Global Moran’s I tool was used to determine the overall pattern and trend of the data. The geographic variation of clustered IPV help seeking behaviour prevalence was assessed using Z-score values and *p*-values. A positive Z score indicates high help seeking behaviour prevalence clustering and a negative Z score indicates low clustering. A positive Moran’s I index value indicates a tendency toward clustering, while a negative Moran’s I index value indicates a tendency toward dispersion [31].

A hot spot analysis was then conducted utilizing the Hot Spot Analysis (Getis-Ord Gi*) tool in ArcGIS [32]. Hot spot analyses display high or low clusters of help seeking behaviours. The hot spot analysis image conveyed that there are areas within Ethiopia with high spatial clustering or better IPV help seeking behaviour than would be assumed by pure randomness.

## Results

**Fig. 1:**
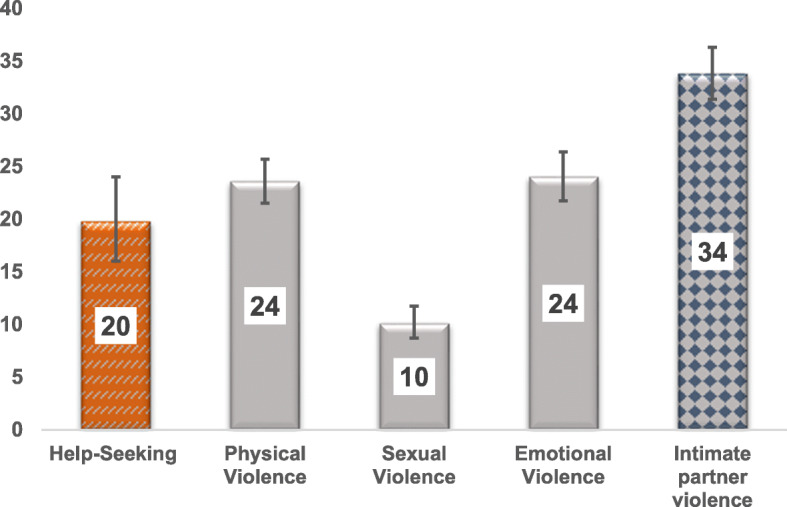
Weighted prevalence and 95% CI of help seeking, physical, sexual, emotional and IPV among married women 15–49 years old, EDHS 2016

The graph below shows the experience of IPV among married women age 15–49 years old was 34%. Both physical and emotional violence were the most reported forms of violence. Among women who had experienced IPV, 20% sought help from a social service provider (Fig. 1). The majority of women (80%) never sought help or told anyone about the physical, sexual or emotional violence they encountered from their partner.

**Fig. 2:**
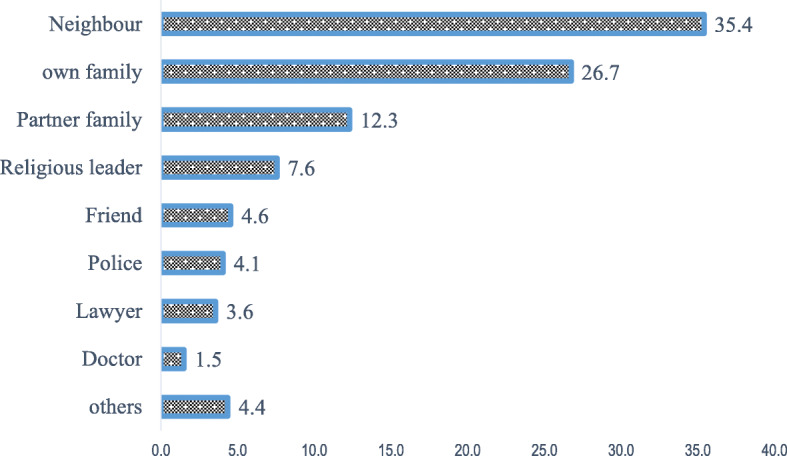
Weighted proportion of married women 15–49 who sought help after IPV experience by sources from which they sought help, EDHS 2016

Below graph (Fig. 2) shows that among a small proportion of women who sought help, the top most common sources were neighbours (35.4%), immediate family members (26.7%) and collaborate family (12.3%). Moreover, only a very small proportion sought assistance from a legal(police (4.1%) or lawyer (3.6%) source).

### Prevalence of help seeking by individual and partner characteristics

The weighted prevalence of help seeking behaviour was higher among married women who had experienced physical 21.61% [17.17, 26.83] and emotional violence 25% [19.8, 31.49]. Additionally, women who attained at least a secondary education, were between the ages of 25–34 years old, were formerly married, and those with access to mass media (TV/radio/magazine) were more likely to engage in help seeking behaviour in Ethiopia (Table 3).

**Table 3:** Weighted proportions (95% CI) of help seeking by individual and partner level characteristics among women aged 15–49 years, EDHS 2016

Characteristics	Options	Frequency	Help Seeking Prevalence % [95% CI]	*P*-Value
Physical violence	No	461	9.0[5.15,15.5]	0.002
	Yes	962	21.6[17.17,26.83]	
Sexual violence	No	1065	19.1 [14.46,24.86]	0.681
	Yes	358	20.8 [14.8,28.49]	
Emotional violence	No	370	9.8[6.47,14.76]	< 0.001
	Yes	1053	25.2 [19.8,31.49]	
Women factors				
Age (Years)	15–24	295	10.0 [6.156,15.70]	0.003
	25–34	610	24.3[18.4,31.25]	
	35–49	518	20.5 [14.97,27.41]	
Women education	Secondary and above	839	34.4[21.08,50.72]	0.030
	Primary	413	15.1[10.75,20.81]	
	No education	171	20.0[14.88,26.27]	
Women religion	Muslim	510	18.2[11.73,27.16]	0.510
	Orthodox	589	22.2[16.38,29.34]	
	Other (Catholic/traditional)	324	17.8[12.02,24.2]	
Women working status	working	567	21.6[16.7,27.53]	0.460
	Not working	856	18.7[13.72,25.12]	
Women’s age at first marriage (years)	18 and above	541	17.2[12.63,22.93]	0.190
	< 18	882	21.2[16.51,26.59]	
Number of union/partner women have	One	1145	20.12 [15.56,25.62]	0.710
	More than One	278	18.5[12.3,26.81]	
Current marital status	Currently married	1190	18.2[14.19,22.94]	0.041
	Formerly married	233	27.4[19.05,37.62]	
Woman ever drank alcohol	No	886	17.6[13.15,23.08]	0.180
	Yes	537	23.3[16.81,30.93]	
Access to communication media	Yes	395	23.5[15.91,33.22]	0.020
	No	1028	18.7[14.11,24.27]	
Partners variables				
Age of partner (years)	15–24	36	10[2.845,28.72]	0.003
	25–35	313	11.2[7.336,16.8]	
	36–50	441	22.9[16.52,30.95]	
	50+	333	25.4[19.12,32.8]	
Duration of marriage (years)	0–4	221	10.4[5.827,17.99]	0.008
	5–9	277	15.3[9.852,23.1]	
	10 and above	925	23.5[18.21,29.79]	
Partner education	Secondary and above	541	13.2[6.78,24.04]	0.230
	Primary	506	15.7 [11.49,20.98]	
	No Education	376	20.9 [14.66,28.89]	
Husband working status	employed	1084	17.1[13.23,21.85]	0.015
	unemployed	339	27.8[19.71,37.74]	
Number of children	0–2	198	15.5[11.03,21.34]	0.263
	3 to 5	427	21.2[15.84,27.88]	
	5+	553	23.3[14.8,34.65]	
Husband has controlling behaviour	No	334	10.5[6.274,17.13]	0.005
	Yes	1089	22.1[17.49,27.47]	
History of woman’s father being physically violent towards her mother	No	803	18[14.05,22.82]	0.301
	Yes	620	21.7 [15.8,28.94]	
Partner drank alcohol	No	856	15.4[10.33,22.39]	0.017
	Yes	567	25.2[20.33,30.88]	

Moreover, there was a correlation between the partner characteristics of married women and help seeking behaviour. The help seeking behaviour of married women was positively linked to having an older partner, increased number of years of cohabitation, unemployment, alcohol consumption, and partners who exhibit controlling behaviour (Table 3).

### Prevalence of help seeking by community and social characteristics

As presented in Table 4, the proportion of help seeking behaviour was highest amongst women residing in urban settings 36.5% [26.24,48.16], those who were from the highest wealth index 34.5% [23.61,47.39], and those women who had the skill to make decisions about their lives 25% [18.23,33.15]. In terms of regional variation, married women from Addis Ababa (42%), Somali (27%) and SNNP (23%) were more likely to seek help for IPV. In contrast, married women from Benishangul-Gumuz (5.5%), Afar (7.8%) and Harari (10%) were unlikely to report help seeking behaviour when they encounter violence from a partner.

**Table 4:** Weighted Prevalence of help seeking by community and societal level among married women aged 15–49 years, EDHS 2016

Characteristics	Options	Frequency	Help-Seeking Prevalence %[95%CI]	*P*-value
Community Factors				
Place of residence	Urban	335	36.5[26.24,48.16]	< 0.001
	Rural	1088	17.0[12.95,22.07]	
Geography (region)	Tigray	162	20.3[12.56,31.1]	0.128
	Afar	81	7.8[2.43,22.21]	
	Amhara	186	19.2[13.54,26.58]	
	Oromia	253	17.6[11.14,26.7]	
	Somali	47	28.6[15.1,47.31]	
	Benishangul-Gumuz	134	5.5[1.93,14.86]	
	SNNP	166	23.8[16.78,32.68]	
	Gambella	120	13.3[7.90,21.65]	
	Harrari	109	10 [4.56,20.93]	
	Addis Ababa	78	42.2[28.76,56.94]	
	Dire Dawa	87	18.6[10.3,31.29]	
Societal factors				
Wealth index	rich	634	34.5[23.61,47.39]	0.003
	middle	229	16.3[10.65,24.06]	
	Poor	352	15.7[10.72,22.5]	
Decision making Power	Full power	364	25[18.23,33.15]	0.204
	Considerable power	253	21[12.48,33.17]	
	No power	806	17.0[12.71,22.45]	
Attitudes of wife beating	not justified	492	24.8[16.89,34.98]	0.110
	justified	931	17.6[13.74,22.27]	
Overall		1423	19.75 [15.9;24.3]	

### Multivariable analysis

The hierarchical logistic regression model was fitted at four levels. In the final model, some of the variables that were significant in the crude association remained significant in the final model. In the final model, higher odds of help seeking behaviour was linked to physical violence, woman’s educational attainment, partner’s unemployment, controlling behaviour, partner’s alcohol consumption, regional variability, and women of high wealth index (Table 5).

**Table 5:** Multivariable analysis of help seeking fitted by four level covariates among married women aged 15–49 years after experienced IPV, EDHS 2016

Help Seeking factors	Option	COR	AOR	[95% CI]	*P*-value
Physical violence	No	1.00	1.00		
	Yes	2.76[1.42; 5.41]	3.22	[1.12, 9.20]	0.029*
Sexual Violence	No	1.00	1.00		
	Yes	1.12[0.66; 1.87]	1.36	[0.75, 2.44	0.307
Emotional violence	No	1.00	1.00		
	Yes	3.11[1.76; 5.36]	1.96	[0.92, 4.20]	0.082
Maternal education	No education	1.00	1.00		
	Primary	0.71[0.18; 0.43]	0.16	[0.03, 0.82]	0.026*
	Secondary and above	2.10[0.06; 0.98]	0.21	[0.04,1.21]	0.080
Women drink alcohol	No	1.00	1.00		
	Yes	1.42[0.83; 2.34]	0.98	[0.43, 2.21]	0.952
Having access any means of communication	Yes	1.00	1.00		
	No	0.75[0.41; 1.37]	1.62	[0.61,4.26]	0.329
Age of partner	15–24	1.00	1.00		
	25–35	0.1.2 [0.29; 4.67]	0.51	[0.12, 2.20]	0.367
	36–50	2.7 [0.69; 10.8]	0.91	[0.21, 3.84]	0.894
	50+	3.12[0.80; 12.13]	0.75	[0.15, 3.89]	0.736
Number of children	0–2	1.00	1.00		
	3 to 5	1.5[0.84; 2.5]	2.07	[0.94, 4.52]	0.069
	5+	1.7[0.84;3.30]	2.21	[0.85, 5.70]	0.103
Husband working status	Employed	1.00	1.00		
	unemployed	1.9[1.12; 3.11]	3.71	[1.29, 10.65]	0.015*
Partner education	Secondary and above	1.00			
	Primary	1.22[0.54; 2.76]	1.96	[0.50, 7.63]	0.331
	No Education	1.74[0.75; 4.03]	4.09	[0.96, 17.37]	0.056
Partner’s controlling behaviour	No	1.00			
	Yes	2.4[1.30; 4.51]	3.24	[1.45, 7.22]	0.004*
History of woman’s father being physically violent towards her mother	No	1.00			
	Yes	1.3[0.81; 1.95]	1.15	[0.65, 2.05]	0.626
Partner ever drinks alcohol	No	1.00			
	Yes	1.92[1.12; 3.08]	2.45	[1.23, 4.86]	0.011*
Place of residence	Urban	1.00			
	Rural	0.34[0.20; 0.63]	1.67	[0.43, 6.49]	0.456
Region/Geography	Tigray	1.00			
	Afar	0.33[0.08; 1.2]	0.54	[0.06, 4.65]	0.576
	Amhara	0.93[0.46; 1.90]	2.68	[0.82, 8.74]	0.101
	Oromia	0.840[.38; 1.83]	1.76	[0.53, 5.87]	0.354
	Somali	1.56[0.58;4.23]	4.70	[0.98, 22.57]	0.054
	Benishangul-Gumuz	0.23[0.07; 0.79]	0.23	[0.02, 2.61]	0.233
	SNNP	1.22[0.59; 2.52]	4.16	[1.20, 14.48]	0.025*
	Gambella	0.6[0.26; 1.37]	1.71	[0.43, 6.84]	0.444
	Harrari	0.44[0.15; 1.23]	1.97	[0.36, 10.88]	0.436
	Addis Ababa	2.86[1.25; 6.54]	4.76	[0.84, 26.99]	0.078
	Dire Dawa	0.89[0.36; 2.19]	3.02	[0.62, 14.76]	0.172
Wealth index	poor	1.00			
	middle	1.04[0.53; 2.0]	0.75	[0.37, 1.53]	0.430
	Rich	2.8[1.41; 5.64]	4.13	[1.29, 13.20]	0.017*
Decision making	Full power	1.00			
	Considerable power	0.83[0.42; 1.54]	1.45	[0.51, 4.17]	0.488
	No power	0.62[0.36; 1.04]	0.85	[0.34, 2.15]	0.734
Wife beating	not justified	1.00			
	justified	0.65[0.37; 1.12]	1.07	[0.56, 2.07]	0.836

* Significant covariates at *P*< 0.05

For instance, women who experienced physical violence were three times more likely to seek help as compared to those who did not experience physical violence. Married women who attained a primary education had 29% higher odds of help seeking behaviour as compared to women with no education. Additionally, women with partners who exhibit controlling behaviour and consume alcohol had higher odds of reporting help seeking behaviour. Women from urban and higher economic wellbeing settings, such as Addis Ababa, had higher odds of seeking help.

### Geographic variation and spatial clustering of help seeking behaviour among married women who experienced IPV

**Fig. 3:**
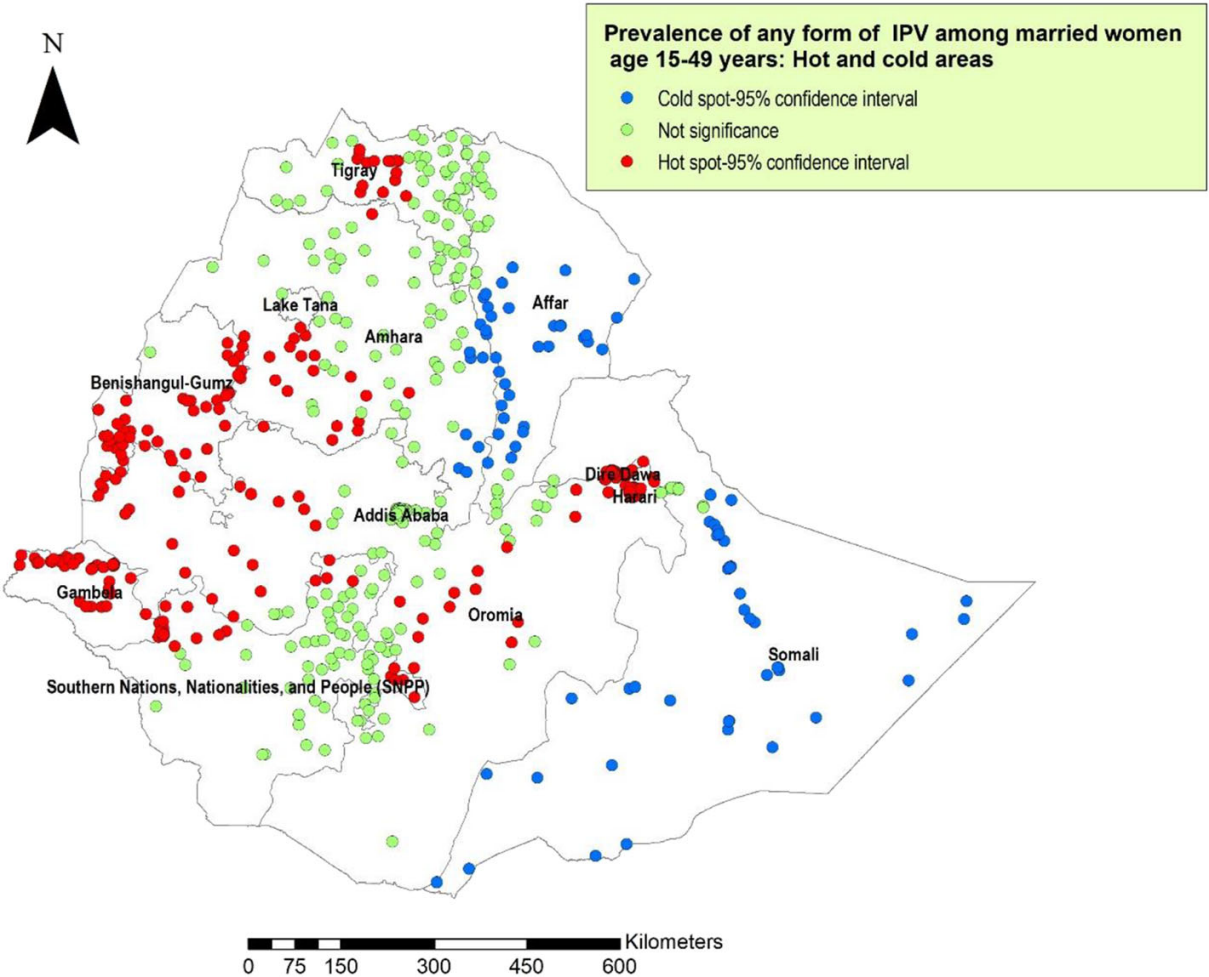
Map of prevalence of IPV among married women age 15–49 years in Ethiopia; EDHS 2016 (additional Fig. 3)

In this study, low prevalence or cold spot areas of IPV were reported in Somalia, Afar, and parts of the Eastern Amhara region. High IPV spots were observed in Western and Central Oromia, Western Amhara, Central Tigray, Eastern Benishangul-Gumuz, Gambella, Dire-Dawa and Harari (Fig. 3). The spatial distribution of IPV showed significant geographic variation among regions in Ethiopia (Moran’s I index =3.15; Z score=47.3; *P*< 0.001).

**Fig. 4:**
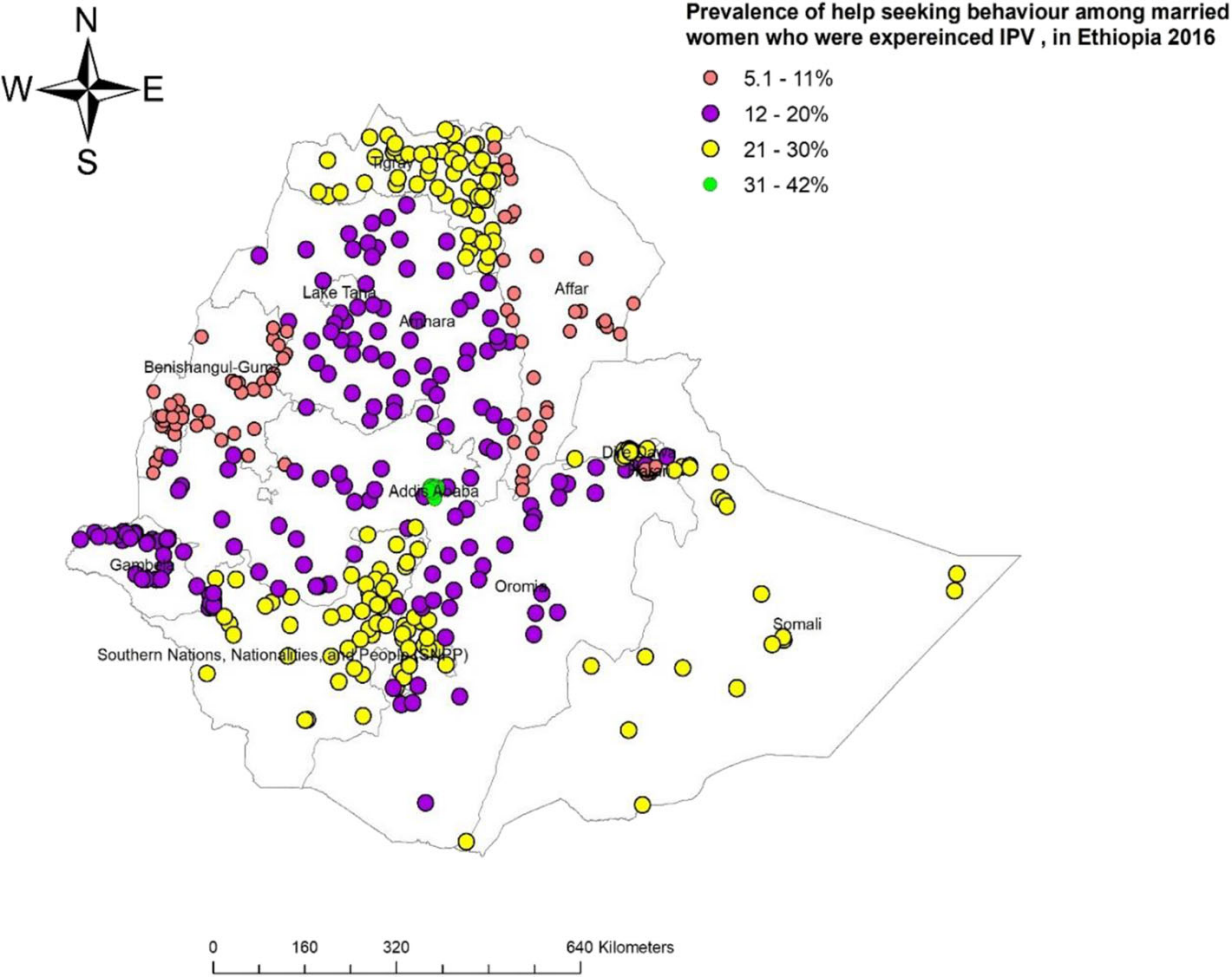
Map of prevalence help seeking behaviour among married women age 15–49 years, EDHS 2016 (additional Fig. 4)

The prevalence of reported help seeking behaviour was high (42%) in Addis Ababa while the lowest was in the developing regions of Ethiopia, especially in Afar, Gambella and Benishangul Gumuz regions (Fig. 4).

**Fig. 5:**
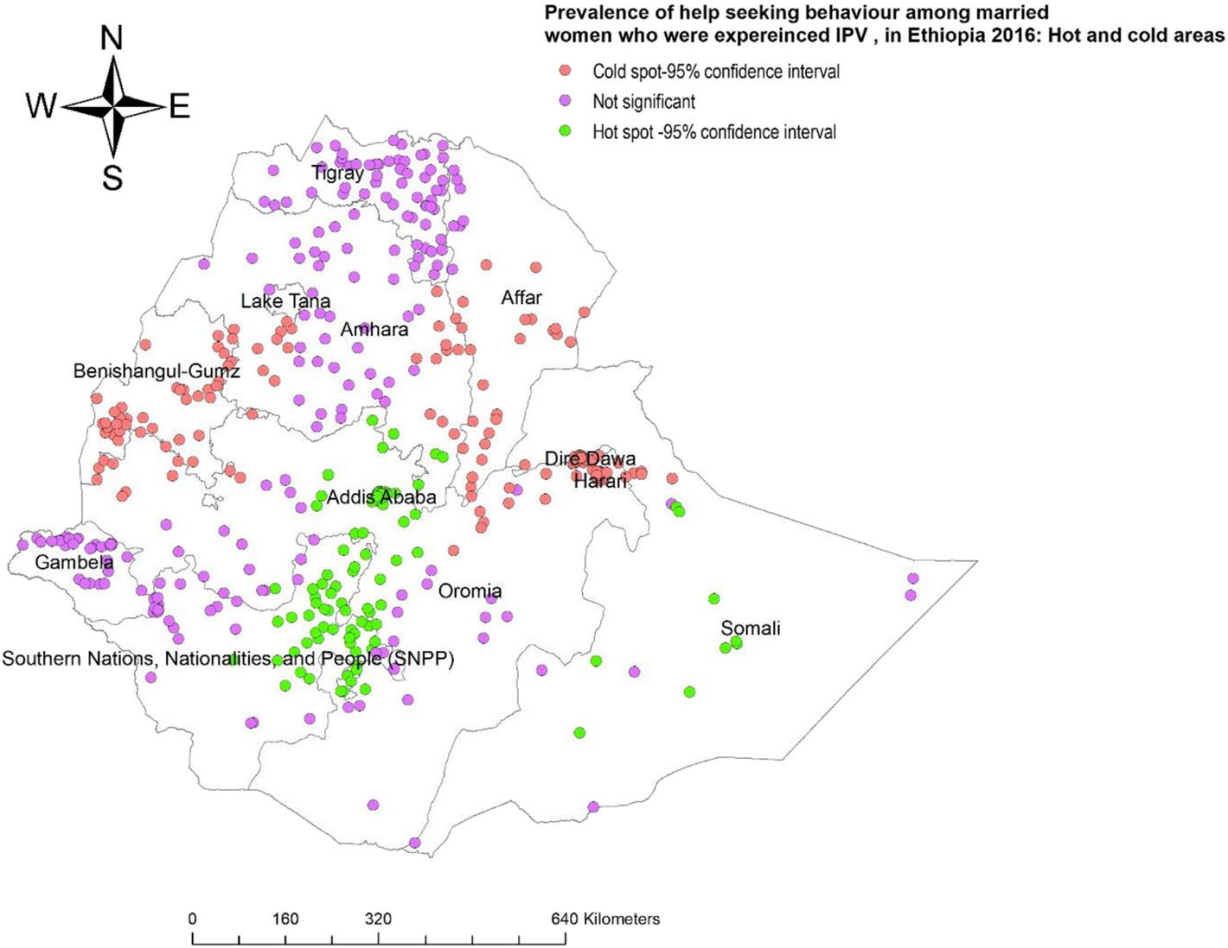
Map of hot and cold areas of help seeking behaviour among married women age 15–49 years old, EDHS 2016 (additional Fig. 5)

The Getis-Ord Gi* statistics showed that the prevalence of reported help seeking behaviour among women had statistically significant variation among regions in Ethiopia. Hot spots of help seeking behaviour among married women who experienced IPV were clustered predominantly around Addis Ababa, Northeastern SNNPR, Central Oromia and Somali. In contrast, Benishangul-Gumz, Eastern and Western Amhara, Harari, and Afar were cold spots, despite high prevalence of IPV in these regions (Fig. 5).

## Discussion

The aim of this study was to identify any geographical variation in help seeking behaviour among married women ages 15–49 years old in Ethiopia and to investigate its associated factors using 2016 EDHS survey data.

Overall, a low prevalence of reported help seeking behaviour in Ethiopia was identified among married women who experienced IPV. This was consistent with other studies conducted in similar settings including in Ethiopia and other SSA countries [2, 33, 34]. It is important to note that the majority of the women who reported help seeking behaviour were seeking support from informal sources such as neighbours, families, or friends. This indicates that IPV in Ethiopia is commonly managed informally at the interpersonal and community levels. Due to the limited training and capacity of informal sources, IPV remains high and in need of intervention. The low prevalence of help seeking behaviour might be related to the socially accepted dominance of husbands over their households, including their spouses. This unequal distribution of power and status in domestic relationships is an important indicator of the dominant cultural and social norms of Ethiopian society [2, 35–39]. Moreover, low prevalence may be influenced by the disparate socio-demographic characteristics of women in Ethiopia, such as low educational attainment. Other factors associated include the type of violence experienced and partner related characteristics, such as controlling behaviour, alcohol consumption, employment status, and wealth of the family. This is consistent with studies conducted in various areas [40–42].

Generally, the prevalence of help seeking behaviour in Ethiopia was very low at 19.75%. The use of spatial analysis showed that the phenomenon was heterogeneous in the various regions of Ethiopia. It also identified clusters of reported help seeking behaviour among women who experienced IPV, mainly in Addis Ababa, North Eastern SNNPR, Central Oromia, and the Somali regions. In contrast, married women in Benishangul-Gumuz, Gambella, East and Western Amhara and Afar regions had the lowest odds of seeking help when they experienced IPV. The geographical variation of IPV in the regions of Ethiopia might be related with the patriarchal nature of community that promotes male dominance and the deep rooted acceptance of violence among women [2, 18]. Moreover, in some of the regions such as Gambella, Afar and other Western Oromia, there are large number of refuges and internally displaced peoples or conflict and displacement disrupt all aspects of life. In addition, there are many peoples who are travelled to Oromia and Benshangule region and often separated from their family and loved ones. This refugees may experience significant distrust and discrimination in the host community of the refuges and migrants confidence to have help seeking behaviour due to fear to new environment and lack of the confidence the access the services [8, 18, 23].

Moreover, the decision to seek help, from either a formal or informal source of support, depends how victims problematize their abusive situation. The findings of this study indicate that women experiencing physical violence is the most significant reason to seek help. This might be attributed to the severity of the injury and pain which may require immediate need of services or support. Moreover, culturally, it is considered less shameful to report IPV to family rather than a formal source. This may be why it was more common for women to report seeking help from informal sources [40, 43].

Attaining a secondary or above education was found to be the most important factor to enhancing help seeking behaviours of women and reducing the occurrence of IPV. In this study, the odds of reporting help seeking behaviour was higher amongst women who have a secondary or above education compared to women who have no education. Studies indicate that education informs women to better be able to identify and mitigate IPV. It also increases the help seeking behaviour of women [37, 44–47]. Lower levels of education attainment might be related to a lack of understanding around the mitigation measures, limited exposure to legal support, and increased acceptance of traditional beliefs which are harmful to women. Moreover, it might also be correlated to other factors such as resources and decision-making power [48]. Health behaviour theory demonstrates that education (increased knowledge and awareness) is a strong means for improved behaviour [37, 49, 50]. Education is a way of informing people of the normative underpinnings of a society and can expose them to global discourses rejecting IPV and encouraging help seeking behaviour [3, 51].

This study shows that partner characteristics are important predictors for women seeking help in response to IPV. More specifically, a partners’ employment status, controlling behaviour, and alcohol consumption were strongly associated with reported help seeking behaviour among married women who experienced IPV in Ethiopia [52].

The interdependence between economic dependence of women and low help seeking behaviour is well documented in various studies [36, 53, 54]. When the partners of women experiencing IPV were unemployed, the likelihood of women reporting the violence was doubled. This suggests women experiencing IPV may leave a dangerous relationship when there is less economic dependence on their partners [55–57].

According to feminist theory, controlling behaviour within relationships is related to unequal social distribution of power, which is rampant in patriarchal societies. This revealed the societal-level power differences within societies that work directly or/and indirectly to endorse a male-dominated social order and family structure. This often result in men exercising power and control over women in several ways, one of which is IPV and the likelihood of help seeking controlled [58, 59]. In contrast, this study found that women with partners exhibiting controlling behaviour were more likely to look for help from formal or informal sources. Although additional research is needed, these findings may indicate that the controlling behaviour of an intimate partner and the use of physical and emotional threats can result in such adverse effects that increase the help seeking behaviour of women [58, 60]. As a result, focusing more attention on understanding the associated factors of controlling behaviour of partners may help to design targeted interventions increase the help seeking behaviour of women.

The strong link found between partner alcohol consumption and increased help seeking behaviour may be attributed to the potential for alcohol to affect the thinking, perceptions and risk-taking of male partners; this may create a fear or increased violence in women which leads to reporting the violence to someone who can provide support [61, 62].

Additionally, this study revealed wealth increases the odds of help-seeking behaviour among women in Ethiopia. This is consistent with previous research which suggests that women who earn higher incomes or who are financially independent are more likely to seek help [63]. Women who live in poverty or low income families are less likely to seek help [64, 65]. Overall, this influence of wealth on help seeking behaviour may be because access to financial resources widens the range of support and services available.

### Implication for programming and policy

Findings reported in this study provide vital evidence to inform programs and policy, and to guide investment in woman’s health to prevent and reduce the consequences of IPV in Ethiopia, in alignment with the 2030 SDG five target that includes target to eliminate all forms of violence against all women and girls including trafficking, sexual and other types of exploitation and SDG 16 target related to significantly reduce all forms of violence and related death rates everywhere and to end abuse, exploitation, trafficking and all forms of violence against children. This study provides insight to the required response to women experiencing IPV by identifying the geographical hot spots in Ethiopia and associated factors with help seeking behaviour. Governmental policy should prioritize the prevention of IPV and increase the demand for formal services by women, especially given the high prevalence of IPV and low help seeking behaviours in all regions of Ethiopia. This strategy needs to be supported by a legal framework to accommodate social support that includes power equality, women’s economic empowerment, and provision of health information and services.

## Strength and limitation of the study

This study used the golden standard measurement for most developing countries (DHS measure), EDHS 2016, a representative survey which is the largest dataset in Ethiopia. The study team used multistage cluster sampling and analysis, and geospatial analysis identify hotspot areas across the country. A limitation of this study is the cross-sectional nature of the study design which may affect causality. Moreover, since the study is self-reported, recall bias may be present and underreported due to fear of stigma and discrimination and cannot be validated by formal and informal sources. Additionally, this study only limited to EDHS questionaries and lacks some variables related to refugees, internally displaced people and many other priorities.

## Conclusions and recommendations

In summary, the findings show that the prevalence of help seeking behaviour among married women who experienced IPV in Ethiopia was very low, although IPV is highly prevalent. The lowest help seeking behaviour prevalence was reported in Benishangul-Gumuz, Western and Eastern Amhara, Gambella and Afar regions. Formal help seeking behaviour in Ethiopia is limited, which poses a challenge to designing effective public health interventions to address IPV. IPV is underreported to formal sources such as police, courts, and other social service institutions. Factors associated with poor help seeking behaviour among married women include low educational attainment, the type of violence experienced, husband controlling behaviour, higher alcohol consumption, partner’s unemployment status, geographical location, and poor wealth level of the household and surrounding community. Moreover, the findings clearly show that intervention requires a multisectoral response including education for capacity building and to create awareness of the women and communities, economic sector for creating business opportunities, health sector to avail services and counsel clients, and legal sector for law enforcement are some of the sector offices that needs to be involved in response to IPV and help seeking. Building the capacity of health care providers in response to psychosocial and gender based violence support. Governmental policies should focus on the prevention of IPV through various means such as increasing demand for women to access formal services (health, legal and social services), if they experienced IPV. Additionally, the help seeking and IPV prevention strategy needs to be supported by a legal framework and law enforcement to accommodate the social support offered in a given community. Future studies should focus on qualitative research that centres an in-depth understanding of gender inequity due to male dominance. Additionally, more research that focuses on interventions to improve women’s reporting of IPV is needed. Moreover, further study that focus on help-seeking behaviours difference in sub-groups such as refugees, ethnic and religious minorities are some research areas.
